# Spatiotemporal Association of Real-Time Concentrations of Black Carbon (BC) with Fine Particulate Matters (PM_2.5_) in Urban Hotspots of South Korea

**DOI:** 10.3390/ijerph14111350

**Published:** 2017-11-06

**Authors:** Sungroul Kim, Sol Yu, Dongmin Yun

**Affiliations:** Department of Environment Health Sciences, Soonchunhyang University, Asan 31538, Korea; solsol0914@gmail.com (S.Y.); balentain19@gmail.com (D.Y.)

**Keywords:** black carbon, PM_2.5_, diesel engine emissions, spatiotemporal distribution, urban air pollution

## Abstract

We evaluated the spatiotemporal distributions of black carbon (BC) and particulate matters with aerodynamic diameters of less than 2.5 m (PM_2.5_) concentrations at urban diesel engine emission (DEE) hotspots of South Korea. Concentrations of BC and PM_2.5_ were measured at the entrance gate of two diesel bus terminals and a train station, in 2014. Measurements were conducted simultaneously at the hotspot (Site 1) and at its adjacent, randomly selected, residential areas, apartment complex near major roadways, located with the same direction of 300 m (Site 2) and 500 m (Site 3) away from Site 1 on 4 different days over the season, thrice per day; morning (*n* = 120 measurements for each day and site), evening (*n* = 120), and noon (*n* = 120). The median (interquartile range) PM_2.5_ ranged from 12.6 (11.3–14.3) to 60.1 (47.0–76.0) μg/m^3^ while those of BC concentrations ranged from 2.6 (1.9–3.7) to 6.3 (4.2–10.3) μg/m^3^. We observed a strong relationship of PM_2.5_ concentrations between sites (slopes 0.89–0.9, the coefficient of determination 0.89–0.96) while the relationship for BC concentrations between sites was relatively weak (slopes 0.76–0.85, the coefficient of determination 0.54–0.72). PM_2.5_ concentrations were changed from 4% to 140% by unit increase of BC concentration, depending on site and time while likely supporting the necessity of monitoring of BC as well as PM_2.5_, especially at urban DEE related hotspot areas.

## 1. Introduction

According to an assessment by the World Health Organization’s (WHO) International Agency for Research on Cancer (IARC), outdoor air pollution, especially particulate matter, is carcinogenic to humans (Group 1). Furthermore, IARC reported that countries can reduce the burden of disease from stroke, heart disease, lung cancer, and respiratory diseases such as asthma by reducing air pollution levels [1]. Diesel engine emissions (DEE), known to be a source of carcinogens contained in outdoor air, is produced as a by-product of incomplete diesel fuel combustion [2]. Particulates with aerodynamic diameters of less than 2.5 m (PM_2.5_) are a mixture of organic and inorganic components, including carbon. In rural and urban areas of central Europe, the contribution of elementary carbon (EC), to PM_2.5_ was found to be 5% and 14%, respectively. At the curbside, elemental carbon contributed up to 21% of the total PM_2.5_ concentrations [1].

Black carbon (BC), carbonaceous component of particulate matter that absorbs all wavelengths of solar radiation, often called equivalent black carbon (eBC) to clarify that what is being measured may not be exactly 100% BC [3,4], is widely used as an indicator of DEE, and short-lived climate forcer, and known as a better indicator of harmful particulate substances from combustion sources (especially traffic) than undifferentiated PM mass. It has a higher association with the incidence of respiratory or cardiovascular diseases per unit (μg/m^3^), as compared to PM_2.5_ or PM_10_ [5]. Because of the environmental health risks associated with BC, understanding exposure level to BC and development of the regulation to reduce total BC emissions is a high priority among global stakeholders, including the United States Environmental Protection Agency [6] as well as those in South Korea.

In South Korea, before the application of Euro 6 emission standards [7], new diesel vehicle registrations were 31.5% of the total number of vehicles in 2011 and 47.1% in 2014. Therefore, concentrations of DEE-related BC and PM_2.5_ in cities is expected to be increased with the number of diesel vehicle registrations. Several previous studies reported temporal and spatial variations of PM_2.5_ concentrations or characteristics of PM_2.5_ measured at national air quality monitoring sites or a PM_2.5_ monitoring supersite South Korea [8,9,10].

However, using air pollution monitoring site data, understanding air quality, especially BC and PM_2.5_ at DEE hotspots and evaluation of the association of BC with PM_2.5_ concentrations are likely limited due to the lack of monitoring resolution. Assessing the hotspot-specific spatial variability of DEE-related air pollutants with increased resolution provides key information to estimate personal exposure and to identify heavily polluted areas. Mobile sampling techniques have been used because of their measurement ability that can be deployed on motor vehicles [11,12,13,14]. However, because mobile sampling techniques monitor air pollution levels while moving, they are unsuitable for simultaneous capturing the fine spatiotemporal patterns at multiple places and for pinpointing periods of the day that are most detrimental to human health.

Simultaneous measurements using portable real-time monitors can provide a substantial improvement on establishing a pollution monitoring network, as compared to traditional approaches using measurements from a small number of fixed air monitoring stations. Specifically, such simultaneous real-time monitoring has proven very effective when a pollutant has a relatively short lifespan, and is subject to meteorological conditions [15,16]. In this study, we evaluate the spatiotemporal distribution of BC and PM_2.5_ concentrations from urban DEE hotspots and associate their concentrations with those of adjacent residential areas using data from portable, real-time pollution monitors.

## 2. Materials and Methods

### 2.1. Sampling Locations

**Figure 1:**
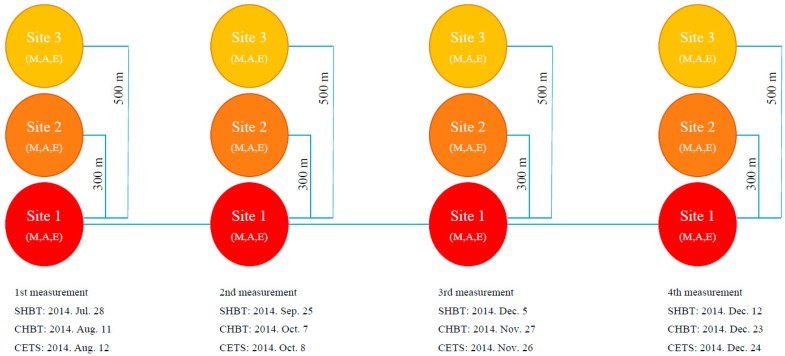
Sampling design applied for simultaneous PM_2.5_ and BC measurement at three different diesel transportation hotspots and their adjacent residential areas (300 m or 500 m away from its hotspot) selected in South Korea. SHBT: Seoul Highway Bus Terminal, CHBT: Cheonan Highway Bus Terminal, and CETS: Cheoanasan Express Train Station; M: Morning (*n* = 120 per day and Site), A: Afternoon (*n* = 120), E: Evening (*n* = 120).

Concentrations of BC and PM_2.5_ were measured at the entrance gate of the Seoul Highway Bus Terminal (SHBT) [37.506496, 127.003856], Cheonan Highway Bus Terminal (CHBT) [36.820977, 127.156413], and Cheoanasan Express Train Station (CETS) [36.795011, 127.104408], between July and December 2014. Measurements were conducted simultaneously at the gate of each location (Site 1) and at randomly selected adjacent residential areas, apartment complexes near major roadways, which were located with the same direction of 300 m (Site 2) and 500 m (Site 3) away from Site 1. Measurements were recorded on 4 days at each site to evaluate the association of concentrations of BC or PM_2.5_ at diesel transportation hospots with those of adjacent residential urban areas (Figure 1). Four-day measurement results for each site were used because our study was conducted to evaluate the contribution of BC to PM_2.5_ concentrations rather than the site representative BC and PM_2.5_ concentration levels. The SHBT and CHBT are located in Seoul and Cheonan, respectively. Although buses circulating the inner city use liquefied petroleum gas (LPG), highway buses use diesel in South Korea. CETS is also located in Cheonan and almost all national train lines (the south west bound and south east bound from Seoul) intersect at this station. On each sampling day, measurements were taken thrice daily; during the morning rush hour (from 07:00 to 09:00), evening rush hour (from 18:00 to 20:00), and noon (from 12:00 to 14:00).

### 2.2. PM_2.5_ and BC Measurement

To ensure the simultaneous measurement of PM_2.5_ and BC, real time monitors were used; the SidePak AM 510 (TSI Inc., Shoreview, MN, USA; PM_2.5_) and AE51 (Aethlabs, San Francisco, CA, USA; BC) measured by a light scattering and optical absorption method, respectively. PM_2.5_ and BC concentration data were logged every minute during the 2-h recording period. The flow rates for the AM510 and AE51 devices were 1.7 L/min and 0.1 L/min, respectively. For this study, after we confirmed the high correlation between measurements of multiangle absorption photometer (MAAP) (Thermo fisher scientific, Waltham, MA, USA) and AE51 with slope of 0.92 and the coefficient of determination of 0.9 [17], we applied mass absorption efficiency (MAE) of 5.0 (m^2^/g) for AE51 operated with wavelength of 880 nm which was a similar result reported by Cheng and Lin (2013) [18].

A conversion factor of 0.4 was applied to the real time PM_2.5_ concentrations obtained from AM510 using a light scattering technique to convert gravimetric method based mass concentrations [19]. The BC monitoring device (AE51) provided negative values when the difference in light attenuation between two consecutive readings was negligible. Therefore, we checked the difference in light attenuation between consecutive readings and then eliminated pairs of BC concentrations associated with a light attenuation differential below 0.05. BC data was then averaged across time intervals associated with changes in incremental light attenuation of 0.05 with a sampling interval of 1 min. Less than 1% of the data showed negative values in our study.

### 2.3. Statistical Analysis

The PM_2.5_ and BC concentration variations between the primary and secondary measurement sites, as well as differences between the three times of the day were compared using the Mann-Whitney or Kruskal-Wallis tests, respectively. Spearman correlation coefficients were obtained measurement by sampling site and time. Log-transformation was conducted for dependent variables in regression analysis to account for the right-skewed distribution of PM_2.5_ or BC concentration.

First, spatial relationships of measurement between Sites 1 and 2 or Sites 1 and 3 were evaluated by developing univariate linear regression line and comparing slopes interpreted as % change of dependent variable by unit increase of independent variable [20] and coefficients of determination providing the information of simultaneous source contribution of hotspot (Site 1) to the two adjacent locations (Site 2 or 3) with time matched PM_2.5_ data. Second, we evaluated with time matched BC concentrations in the same way. The temporal relationships between the sites were examined for the three daily sampling periods; morning rush hour, noon, and evening rush hour by comparing the variation of PM_2.5_ or BC concentrations in each site. Finally, we evaluated the impact of a unit increase in BC concentration on the PM_2.5_ concentrations for each site. All statistical tests were carried out using SAS, version 9.4 (SAS Institute, Cary, NC, USA).

## 3. Results

### 3.1. Distributions of Overall PM_2.5_ and BC Concentrations

**Figure A1:**
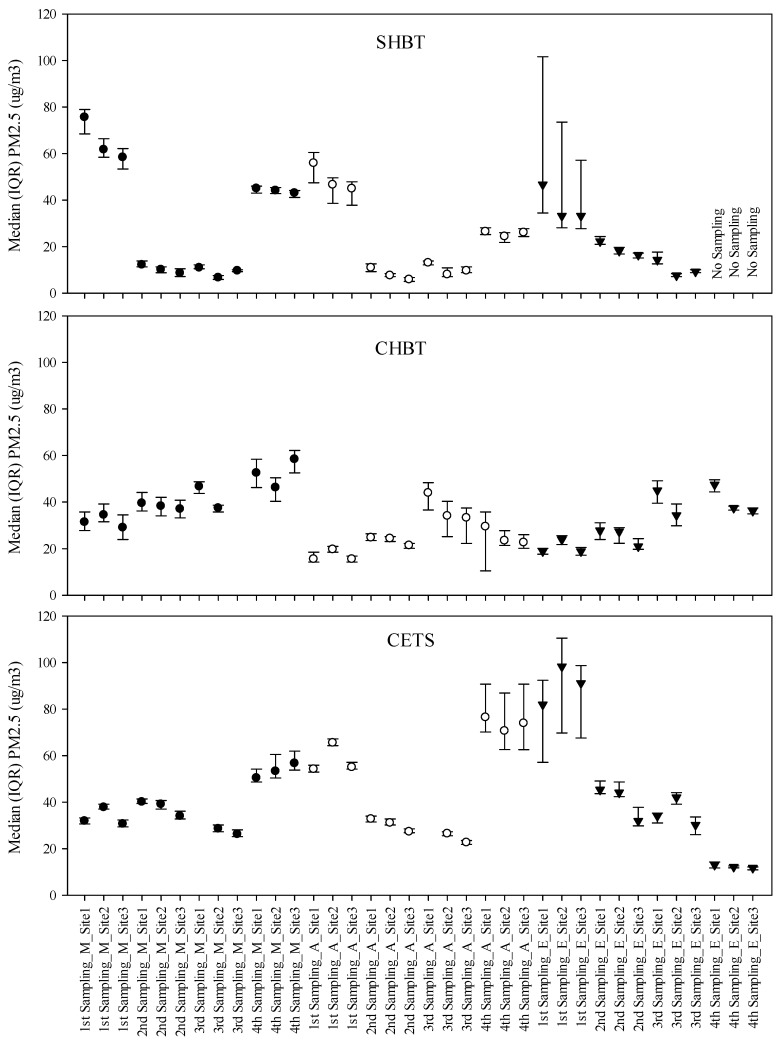
Distributions of PM_2.5_ concentrations (Median (IQR)) of simultaneous measurement for three sites according to sampling place (SHBT, CHBT, CETS), sampling day (1st to 4th trial) and sampling time (Morning, Afternoon, Evening).

**Figure A2:**
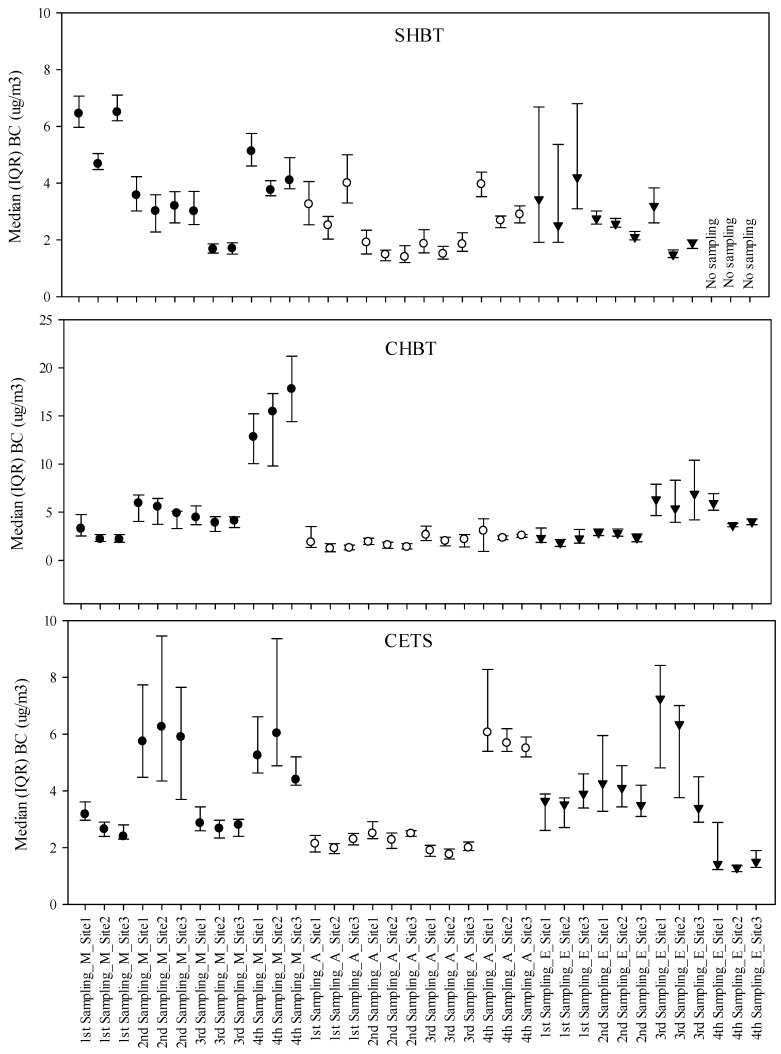
Distributions of BC concentrations (Median (IQR)) of simultaneous measurement for three sites according to sampling place (SHBT, CHBT, CETS), sampling day (1st to 4th trial) and sampling time (Morning, Afternoon, Evening).

Summarized distributions of the PM_2.5_ and BC concentrations (median (Interquartile Range, IQR)) measured simultaneously at Sites 1, 2 and 3 for the SHBT, CHBT, and CETS locations thrice a day are shown in Table 1. The median (IQR) PM_2.5_ concentrations at Site 1 (Figure A1 in Appendix A) ranged from 12.6 (11.3–14.3) to 60.1 (47.0–76.0) μg/m^3^ for SHBT, from 19.7 (16.8–28.1) to 45.6 (35.3–50.4) μg/m^3^ for CHBT, and from 34.0 (31.1–35.3) to 52.9 (33.2–58.6) μg/m^3^ for CETS. Generally, the PM_2.5_ levels at Site 2 (300 m away from Site 1) and Site 3 (500 m away from Site 1), were lower than that of Site 1 by 3.0–39.7% and 4.3–37.5%, respectively. However, the first measurement values for Sites 2 and 3 for the CHBT location and the first and fourth values for Sites 2 and 3 at the CETS location show higher values as compared to Site 1. Median (IQR) BC concentrations at Site 1 (Figure A2 in Appendix A) ranged from 2.7 (2.2–3.4) to 4.6 (2.7–6.5) μg/m^3^ for SHBT, from 2.6 (1.9–3.7) to 6.3 (4.2–10.3) μg/m^3^ for CHBT, and from 2.9 (2.1–5.3) to 5.3 (2.9–6.4) μg/m^3^ for CETS. BC concentrations for Sites 2 and 3 were lower by 0.2–42% or 7.0–35.1%, respectively, as compared to Site 1.

### 3.2. Spatial Relationships of PM_2.5_ and BC Concentrations between Sample Sites

**Figure 2:**
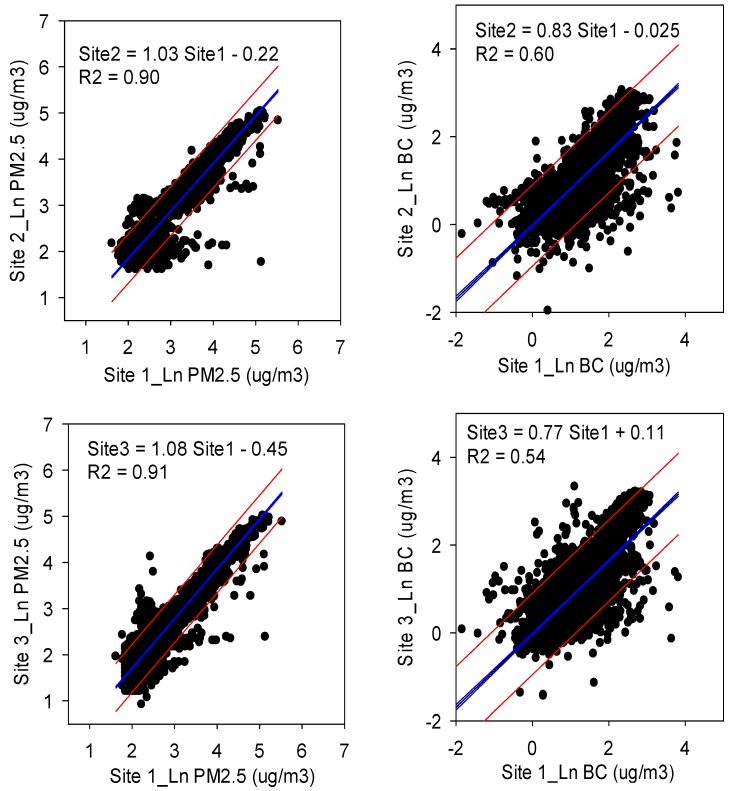
Spatial associations of time matched PM_2.5_ (**Left**) or BC (**Right**) concentrations among sampling sites.

Figure 2 shows the spatial associations of time matched PM_2.5_ and BC measurements between Sites 1 and 2, and between Sites 1 and 3. We observed a strong association of PM_2.5_ concentration values between Sites 1 and 3 as well 1 and 2 (slopes 0.89–0.9, coefficients of determination 0.89–0.96). Conversely, the associations of BC concentration values between sites were relatively weaker (slopes 0.76–0.85, coefficients of determination 0.54–0.72).

### 3.3. Temporal Relationships of PM_2.5_ and BC Concentrations between Sample Sites

The temporal relationships between the three sites were examined for the three daily sampling periods: morning rush hour, noon, and evening rush hour. A summary of the slope interpreted as percent (%) change obtained from regression analyses using temporally matched data is shown in Table 2. For PM_2.5_ concentrations, the temporal distributions were consistent among the three sampling time bins of each Site. However, in the case of BC, we observed that the degree of the variation of BC concentrations for the three time bins were much greater and different depending on sampling time bin.

### 3.4. The Impact of BC Concentration on PM_2.5_ Levels

**Figure 3:**
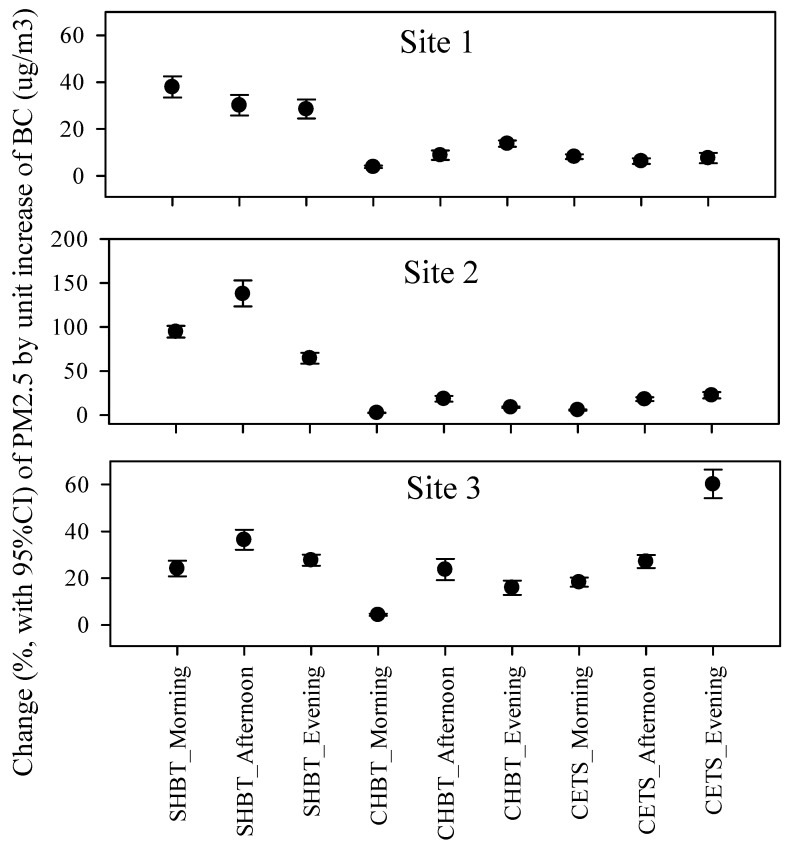
Impact of variation of BC concentration on PM_2.5_ concentrations at each sampling site and sampling time evaluated by the % change of PM_2.5_ concentrations by unit increase of BC (μg/m^3^).

The impact of unit increase in BC concentration on PM_2.5_ concentrations was examined for each sampling place and time (Figure 3). It is apparent that the spatiotemporal impact of BC on PM_2.5_, expressed using a % change to PM_2.5_ concentrations per unit increase of BC concentration (μg/m^3^), differed between the sampling locations and sampling time.

The impact (%) of BC on PM_2.5_ levels was higher at SHBT (Site 1: 38%, 30% and 28% for morning, afternoon and evening; Site 2: 95%, 138% and 64%; Site 3: 24%, 36% and 28%, respectively) as compared to CHBT (8%, 6% and 8%; 6%, 18% and 22%; 18%, 27% and 60%, for Sites 1, 2 and 3, respectively) and CETS (4%, 9% and 14%; 3%, 18% and 9%; 4%, 24% and 16%, for Sites 1, 2 and 3, respectively). From these analyses, we found that coefficients of determination ranged between 0.31 and 0.79 for overall sampling times at SHBT Sites 1, 2 and 3. For CETS and CHBT, the coefficients for the morning, afternoon and evening rush hour period, ranged between 0.40 to 0.81; 0.14 to 0.65; and 0.10 to 0.51, respectively.

## 4. Discussion

Our results showed that overall median concentration levels (8–65 μg/m^3^ for PM_2.5_; 1.8–6.3 μg/m^3^ for BC) were similar or higher, compared to those measured in Brazilian urban areas (5.5–6.0 μg/m^3^ and 2.2–2.4 μg/m^3^, for PM_2.5_ and BC respectively) [21]. However, our levels were lower than results from Dhaka (11–328 μg/m^3^ for PM_2.5_ and 4–48 μg/m^3^ for BC, respectively) [22]. Also, the difference of concentrations of PM_2.5_ or BC at Site 2 or 3, compared to Site 1, ranged −40% to +20% depending on sampling time. Furthermore, we found that even within the same sampling location, the contribution of BC to PM_2.5_ could differ depending on the sampling time by 4% to 140%.

Possible explanations for the variation of PM_2.5_ and BC in urban micro-environment over space and time are heterogeneity in traffic volume and type, driving mode and meteorological conditions. According to Targino et al. (2016) [21] reporting BC and PM_2.5_ concentrations obtained in center area (2.7 km^2^) of midsized city in southern Brazil, lower median BC value in the afternoon, compared to that of morning, were observed with decrease (2.9–4.3%) of share of heavy-duty diesel vehicles (HDDV) in the afternoon. However, at the same time, their study reported that the increase in PM_2.5_ was due to non-exhaust particles and secondary particle formation and growth from the larger number of total vehicles in the afternoon [22,23]. The study also reported that BC concentrations respond to the location of traffic signal (either on flat or inclined terrain); the level of BC at an inclined street junction with an hourly HDDV rate of 80–100 vehicle h^−1^ was two times higher than the BC concentration observed at a traffic signal located at a flat junction with the same traffic type and density while PM_2.5_ concentrations showed no association with the location of traffic light.

Vilcassim et al. (2014) [24] reported that BC concentrations, even in the same New York City subway station, varied depending on the train type. They found that the BC concentration dramatically increased to more than 100 μg/m^3^ when a diesel-powered maintenance train passed through the station, indicating that variations in diesel traffic volume or type could significantly contribute to an elevated BC concentration. Because BC is a good indicator of DEE, it is not surprising that we found spatiotemporal differences in BC while at the same time, relatively highly consistent PM_2.5_ levels at our sampling sites. Local meteorological conditions can affect an alternate way, after calculating hourly mean PM_2.5_ or BC values, we evaluated the impact of BC on PM_2.5_ levels after accounting for wind speed, temperature, relative humidity and wind direction (Table A1). We found that results from univariate regression analysis were similar to those from multivariate regression.

According to Wu and Xia’s study [18] conducted in Beijing, China, during the winter of 2013, overall mass absorption efficiency (MAE) of BC was 4.2 m^2^/g, and BC concentration can be changed by applying a different MAE value as the increase MAE rate of 0.1 m^2^/g per 10%RH obtained from their study. In this study, we used MAE of 5.0 and median (IQR) hourly relative humidity over our sampling dates was 62% (55–79%). Since we conduct this study with random sampling on no rainy day over three seasons, we considered the effect of RH was relatively small; it cannot account for discrepancies in the data variability. To validate our results, after calculating hourly mean PM_2.5_ or BC values, we evaluated the impact of BC on PM_2.5_ levels after accounting for wind speed, temperature, relative humidity and wind direction. We found that results from univariate regression analysis were similar to those from multivariate regression with one-hour data shown in the Appendix A (Table A1).

Our study results were also supported by a domestic study conducted in metropolitan city of Gwangju, South Korea that reported that diurnal patterns in BC exhibited peak concentrations during the morning and evening hours, coinciding with rush-hour traffic [25]. The study also determined that two BC episodes, a high BC concentration level (>10 μg/m^3^) and low PM_2.5_ and SO_4_^2−^ concentrations, and a high BC (>10 μg/m^3^) and high and low PM_2.5_ and SO_4_^2−^, could be attributed to locally produced emissions. Park and Lee’s study supports our finding that the temporal relationship between BC and PM_2.5_ differs depending on short term circumstances.

Conclusively, in our study, consistency of PM_2.5_ concentrations was possibly a result of the existence of suspended particles from tire wear or ultrafine particles from secondary formation of traffic related existing aerosols in urban micro-environment, even though there was large variation of the level of BC within 500 radius due to difference of traffic volume, type of diesel vehicle and driving conditions over sampling site and time.

According to the previous studies, a unit increase in carbon concentration was associated with a 1.45% increase in all-cause mortality [26,27], elevated risk of wheeze (1.45%), shortness of breath (1.41%), and total symptoms (1.30%) [24,28,29]. Such an impact was observed with BC concentration levels between 1 and 5 μg/m^3^, which was similar to the BC levels found in our study. Furthermore, a recent literature review by WHO found that the short-term health effects caused by exposure to BC were more significant, as compared to PM_2.5_ and PM_10_. With consideration of the spatiotemporal variation of BC concentrations and its potential health impacts, our study suggests that regular BC monitoring in addition to PM_2.5_ monitoring in urban diesel exhaust related hotspot areas is important to improve the health outcomes for the urban population in South Korea.

The results from this study should be interpreted with consideration to the limitations of our experimental design. Firstly, four-day measurement results for each site and location were used because our study was conducted to evaluate the contribution of BC to PM_2.5_ concentrations rather than the site representative BC and PM_2.5_ concentration levels. Furthermore, data regarding traffic type or traffic volume was not collected due to the difficulty of distinguishing between diesel and gasoline vehicles in South Korea. Therefore, our results could not provide insight as to the impact of vehicle type on BC or PM_2.5_ concentration levels.

Secondly, despite the potential impact of meteorological conditions on the concentration level of PM_2.5_ or BC, a lack of time matched data for meteorological conditions at the study sites prevented the use of a multivariate analysis at this temporal scale. Therefore, it was difficult to ascribe too much to the impact of meteorological condition.

Thirdly, our data was collected during three 2-h long sampling periods rather than continuously. Therefore, we could not provide daily concentration levels. If sampling was conducted over 24 h, diurnal and nocturnal PM_2.5_ and BC concentration levels could be explained. Using hourly mean PM_2.5_ and BC concentrations calculated from our data, we found the overall impact (95% confidence interval, 95% CI) of a unit increase of BC concentration on PM_2.5_ was 13.0 (6.8–19.5) after accounting for meteorological conditions (Data available on Table A1).

Notwithstanding these limitations, our study demonstrated that the spatiotemporal distribution of PM_2.5_ within a 500 m radius around a diesel emission hotspot was relatively consistent while the distribution of BC varied markedly. The implications of such different patterns between PM_2.5_ and BC in urban micro-environment were potentially significant when we scaled the 500 m radius to the metropolitan city occupied by a significantly larger number of diesel vehicles traveling every day in this country. Additional research is being considered that will build on the results of the current study by refining our methods for traffic classification to include vehicle fuel type (i.e., gas vs. diesel), meteorological conditions of short-term interval as additional explanatory variables in further describing the impact of DEE on air quality.

## 5. Conclusions

Considering the spatiotemporal variation of BC concentrations and their potential health impacts, we consider that our study provides compelling evidence for support of more rigorous policy initiatives aimed at encouraging a complete implementation of new stringent exhaust emission standards for vehicles commuting in the intercity. In addition, we hope this study provides evidence to support the regular monitoring of BC and PM_2.5_ in urban DEE related hotspot sites and its adjacent residential areas.

## Figures and Tables

**Table 1:** Distributions of PM_2.5_ and black carbon (BC) concentrations (Median (IQR)) of simultaneous measurement for three sites according to sampling place and sampling day (1st to 4th trial).

			PM_2.5_						BC					
			Site 1		Site 2		Site 3		Site 1		Site 2		Site 3	
Place	Trail	N	Median (P25–P75)	Ref	Median (P25–P75)	Dif. * (%)	Median (P25–P75)	Dif. * (%)	Median (P25–P75)	Ref	Median (P25–P75)	Dif. * (%)	Median (P25–P75)	Dif. * (%)
SHBT	1st	360	60.1 (47.0–76.0)	100	49.6 (37.0–62.2)	−17.4	47.9 (35.3–58.4)	−20.3	4.6 (2.7–6.5)	100	3.0 (2.2–4.7)	−34.2	5.0 (3.5–6.8)	+9.5
	2nd	360	13.4 (11.3–21.0)	100	10.1 (8.0–16.8)	−24.9	8.4 (6.3–15.1)	−37.5	2.7 (2.2–3.5)	100	2.4 (1.6–2.8)	−11.9	2.1 (1.7–2.7)	−23.3
	3rd	360	12.6 (11.3–14.3)	100	7.6 (6.7–8.4)	−39.7	9.7 (8.8–10.1)	−23.3	2.7 (2.1–3.4)	100	1.6 (1.4–1.8)	−42.0	1.8 (1.6–2.0)	−33.1
	4th	360	26.5 (25.2–28.1)	100	33.4 (24.4–44.1)	+26.2	28.6 (26.0–42.4)	+7.9	4.5 (3.9–5.2)	100	3.1 (2.7–3.8)	−30.4	3.4 (2.9–3.9)	−24.8
CHBT	1st	360	19.7 (16.8–28.1)	100	24.6 (21.0–31.7)	+24.6	18.9 (16.4–24.4)	−4.3	2.6 (1.9–3.7)	100	1.8 (1.3–2.3)	−28.0	1.9 (1.5–2.6)	−26.0
	2nd	360	28.1 (24.4–38.2)	100	26.5 (23.5–34.0)	−5.8	23.1 (21.0–34.7)	−17.9	3.0 (2.2–4.7)	100	2.7 (1.9–4.5)	−9.6	2.1 (1.4–2.9)	−32.2
	3rd	360	45.4 (40.7–48.7)	100	36.1 (30.2–39.5)	−20.4	33.2 (22.3–37.4)	−26.9	4.2 (3.0–6.1)	100	3.4 (2.4–4.8)	−19.7	3.6 (2.6–4.9)	−15.1
	4th	360	45.6 (35.3–50.4)	100	37.0 (27.7–41.6)	−18.9	36.1 (26.0–52.5)	−20.8	6.3 (4.2–10.3)	100	3.6 (2.6–9.8)	−42.3	4.1 (2.7–14.4)	−35.1
CETS	1st	360	52.9 (33.2–58.6)	100	64.7 (39.1–71.0)	+22.3	54.6 (32.3–61.7)	+3.2	3.0 (2.2–3.7)	100	2.5 (2.0–3.2)	−16.0	2.7 (2.3–3.7)	−11.0
	2nd	360	40.3 (32.8–44.9)	100	39.1 (32.8–42.8)	−3.0	31.5 (27.7–34.9)	−21.9	4.2 (2.7–6.2)	100	3.7 (2.5–5.3)	−12.7	3.2 (2.5–4.4)	−23.6
	3rd	360	34.0 (31.1–35.3)	100	28.1 (26.9–39.1)	−17.4	25.2 (23.5–28.6)	−25.9	2.9 (2.1–5.3)	100	2.7 (1.9–3.9)	−7.6	2.6 (2.1–3.2)	−10.5
	4th	360	50.4 (14.3–70.1)	100	53.3 (12.6–67.6)	+5.8	56.7 (12.2–66.8)	+12.5	5.3 (2.9–6.4)	100	5.3 (1.4–6.1)	−0.2	4.4 (1.9–5.4)	−16.5

SHBT: Seoul Highway Bus Terminal, CHBT: Cheonan Highway Bus Terminal, CETS: Cheoanasan Express Train Station, Ref: reference, Dif.: difference of median concentration, compared to Site 1, [(Site 2 or 3–Site 1)/(Site 1) × 100], * *p* < 0.05.

**Table 2:** Impact of unit increase of PM_2.5_ (Top) or BC (Bottom) concentrations of Site 1 on Site 2 or Site 3 for morning rush hour, lunch time, or evening rush hour. All of the values of % changes were statistically significantly different from 0 at the significant level of 0.05. A positive value suggested that % increase of BC or PM_2.5_ concentration at Site 2 or 3 by unit (μg/m^3^) increase of BC or PM_2.5_ concentration at Site 1, respectively.

**PM_2.5_**		**Site 2—by Site1**		**Site 3—by Site 1**		**Site 3—by Site 2**	
		**R^2^**	**% Change**	**R^2^**	**% Change**	**R^2^**	**% Change**
SHBT	Morning	0.78	2.84	0.83	2.63	0.95	3.56
	Afternoon	0.84	3.67	0.78	3.87	0.86	5.02
	Evening	0.72	2.43	0.79	2.12	0.87	2.84
CAETS	Morning	0.82	1.92	0.93	3.05	0.79	2.74
	Afternoon	0.81	1.71	0.90	1.92	0.97	2.22
	Evening	0.85	2.74	0.91	2.63	0.93	2.22
CYHBT	Morning	0.62	1.31	0.80	2.84	0.70	3.98
	Afternoon	0.76	1.71	0.73	2.02	0.85	4.19
	Evening	0.54	1.11	0.73	1.82	0.91	4.39
**BC**		**Site 2—by Site 1**		**Site 3—by Site 1**		**Site 3—by Site 2**	
		**R^2^**	**% Change**	**R^2^**	**% Change**	**R^2^**	**% Change**
SHBT	Morning	0.17	7.25	0.14	9.42	0.65	47.70
	Afternoon	0.27	11.63	0.18	15.03	0.47	66.53
	Evening	0.29	15.03	0.25	16.18	0.55	34.99
CAETS	Morning	0.34	10.52	0.25	6.18	0.68	12.75
	Afternoon	0.33	1.61	0.25	1.01	0.88	25.86
	Evening	0.41	15.03	0.13	6.18	0.34	13.88
CYHBT	Morning	0.37	7.25	0.64	15.03	0.83	15.03
	Afternoon	0.22	0.80	0.24	0.80	0.33	41.91
	Evening	0.23	8.33	0.23	9.42	0.58	24.61

## Appendix A

Table A1 Summary of the hourly meteorological conditions per each sampling place during the sampling sessions. Table A2 Spearman correlation coefficient according to each sampling site and time. Figure A1 distributions of PM_2.5_ concentrations (Median (IQR)) of simultaneous measurement for three sites according to sampling place (SHBT, CHBT, CETS), sampling day (1st to 4th trial) and sampling time (Morning, Afternoon, Evening). Figure A2 distributions of BC concentrations (Median (IQR)) of simultaneous measurement for three sites according to sampling place (SHBT, CHBT, CETS), sampling day (1st to 4th trial) and sampling time (Morning, Afternoon, Evening).

**Table A1:** Summary of the hourly BC and PM_2.5_ concentrations with meteorological conditions per each sampling place during the sampling sessions.

Place	MM	DD	Hour	BC (μg/m^3^)	PM_2.5_ (μg/m^3^)	Temp (°C)	WD	WS (m/s)	RH (%)
SHBT	7	28	7–9	6.18	63.55	24.85	60.00	3.05	77.00
			12–14	2.85	36.18	30.85	100.00	2.00	47.50
			18–20	6.37	79.97	28.75	280.00	3.55	69.00
	9	25	7–9	3.15	10.64	18.20	50.00	1.00	76.00
			12–14	1.89	10.78	26.95	160.00	1.90	44.50
			18–20	2.84	19.14	24.50	280.00	2.70	62.00
	12	5	7–9	2.42	9.98	−9.50	330.00	1.65	42.50
			12–14	1.68	9.88	−3.85	305.00	4.45	36.50
			18–20	2.74	15.53	−5.30	295.00	2.60	49.50
	12	12	7–9	4.37	43.60	−5.50	315.00	1.15	60.50
			12–14	2.92	23.84	−0.50	260.00	2.70	45.50
			18–20	NA	NA	−2.80	280.00	2.45	84.00
CHBT	8	11	7–9	2.96	30.36	19.75	183.45	0.25	90.00
			12–14	1.73	15.23	26.30	313.70	1.40	62.00
			18–20	3.23	19.65	24.75	309.45	0.65	53.00
	10	7	7–9	4.48	34.39	12.00	27.00	0.45	79.00
			12–14	1.69	24.82	21.70	223.40	1.60	36.00
			18–20	2.57	22.51	17.70	141.00	0.70	61.50
	11	27	7–9	3.96	40.78	6.80	231.60	0.75	91.50
			12–14	2.08	30.86	13.90	223.05	1.30	63.00
			18–20	7.21	44.82	10.40	57.80	0.05	80.50
	12	23	7–9	14.73	54.81	−7.20	51.85	0.40	90.00
			12–14	3.10	28.02	1.75	42.85	0.30	60.00
			18–20	4.46	40.89	1.35	23.85	0.45	68.50
CETS	8	12	7–9	2.60	32.72	22.85	80.00	1.30	86.00
			12–14	2.20	56.02	29.65	270.00	1.25	55.00
			18–20	4.12	105.71	27.25	280.00	2.45	61.00
	10	8	7–9	5.85	37.68	13.30	55.00	0.35	84.50
			12–14	2.63	34.72	21.95	65.00	1.20	36.50
			18–20	5.90	51.49	16.20	200.00	1.35	62.00
	11	26	7–9	2.88	28.68	8.45	125.00	2.00	67.50
			12–14	2.94	24.92	10.95	150.00	2.40	62.50
			18–20	6.17	35.76	7.95	150.00	0.60	79.50
	12	24	7–9	6.90	58.95	2.05	205.00	1.65	79.50
			12–14	5.96	86.00	6.30	270.00	2.25	79.50
			18–20	2.28	13.63	1.85	280.00	4.00	55.50

MM: Month; DD: Day; Temp: Temperature; WD: Wind direction; WS: Wind speed; RH: Relative Humidity.

**Table A2:** Spearman correlation coefficient according to each sampling site and time.

	P1	P2	P3	B1	B2	B3	P1	P2	P3	B1	B2	B3	P1	P2	P3	B1	B2	B3
**Seoul Highway Bus Terminal_Morning**							**Afternoon**						**Evening**					
PM_2.5__SITE1 (P1)	1.00						1.00						1.00					
PM_2.5__SITE2 (P2)	0.72	1.00					0.95	1.00					0.88	1.00				
PM_2.5__SITE3 (P3)	0.66	0.83	1.00				0.95	0.94	1.00				0.90	0.97	1.00			
BC_SITE1 (B1)	0.80	0.75	0.78	1.00			0.70	0.64	0.61	1.00			0.36	0.14	0.18	1.00		
BC_SITE2 (B2)	0.69	0.92	0.78	0.70	1.00		0.74	0.77	0.76	0.66	1.00		0.64	0.71	0.67	0.24	1.00	
BC_SITE3 (B3)	0.70	0.87	0.74	0.71	0.84	1.00	0.79	0.78	0.83	0.54	0.75	1.00	0.73	0.80	0.82	0.19	0.57	1.00
**Cheonan Highway Bus Terminal_Morning**							**Afternoon**						**Evening**					
PM_2.5__SITE1 (P1)	1.00						1.00						1.00					
PM_2.5__SITE2 (P2)	0.85	1.00					0.86	1.00					0.94	1.00				
PM_2.5__SITE3 (P3)	0.93	0.89	1.00				0.95	0.93	1.00				0.92	0.93	1.00			
BC_SITE1 (B1)	0.80	0.68	0.68	1.00			0.52	0.47	0.60	1.00			0.27	0.24	0.29	1.00		
BC_SITE2 (B2)	0.82	0.70	0.67	0.84	1.00		0.60	0.54	0.66	0.66	1.00		0.44	0.45	0.48	0.79	1.00	
BC_SITE3 (B3)	0.82	0.67	0.72	0.64	0.71	1.00	0.74	0.65	0.82	0.71	0.76	1.00	0.74	0.76	0.83	0.45	0.61	1.00
**Cheoanasan Express Train Station_Morning**							**Afternoon**						**Evening**					
PM_2.5__SITE1 (P1)	1.00						1.00						1.00					
PM_2.5__SITE2 (P2)	0.74	1.00					0.83	1.00					0.86	1.00				
PM_2.5__SITE3 (P3)	0.93	0.85	1.00				0.82	0.91	1.00				0.85	0.84	1.00			
BC_SITE1 (B1)	0.63	0.55	0.77	1.00			0.58	0.37	0.35	1.00			0.77	0.77	0.71	1.00		
BC_SITE2 (B2)	0.72	0.66	0.88	0.78	1.00		0.38	0.49	0.54	0.19	1.00		0.74	0.76	0.67	0.71	1.00	
BC_SITE3 (B3)	0.76	0.63	0.87	0.75	0.90	1.00	0.42	0.53	0.62	0.25	0.65	1.00	0.66	0.66	0.65	0.69	0.75	1.00

## References

[1] International Agency for Research on Cancer (2016). IARC Monographs on the Evaluation of Carcinogenic Risks to Humans, Outdoor Air Pollution.

[2] International Agency for Research on Cancer (2013). IARC Monographs on the Evaluation of Carcinogenic Risks to Humans, Diesel and Gasoline Engine Exhausts and Some Nitroarenes.

[3] Lack D.A., Moosmüller H., McMeeking G.R., Chakrabarty R.K., Baumgardner D. (2014). Characterizing elemental, equivalent black, and refractory black carbon aerosol particles: A review of techniques, their limitations and uncertainties. Anal. Bioanal. Chem..

[4] Petzold A., Ogren J.A., Fiebig M., Laj P., Li S.-M., Baltensperger U., Holzer-Popp T., Kinne S., Pappalardo G., Sugimoto N. (2013). Recommendations for the Interpretation of “Black Carbon” Measurements. Atmos. Chem. Phys. Discuss..

[5] Janssen N.A., Hoek G., Simic-Lawson M., Fischer P., van Bree L., ten Brink H., Keuken M., Atkinson R.W., Anderson H.R., Brunekreef B. (2011). Black carbon as an additional indicator of the adverse health of airborne particles compared to PM_10_ and PM_2.5_. Environ. Health Perspect..

[6] U.S. Environmental Protection Agency (2016). Mitigating Black Carbon. https://www3.epa.gov/airquality/blackcarbon/mitigation.html.

[7] (2014). Korea Automobile Manufacturers Association, Automobile Statistics, Korean Automobile Industry. http://kama.or.kr/eng/PS/pdf/Total2014.pdf.

[8] Ghim Y.S., Chang Y., Jung K. (2015). Temporal and spatial variations in fine and coarse particles in Seoul, Korea. Aerosol Air Qual. Res..

[9] Cayetano M.G., Hopke P.K., Lee H., Jung J., Batmunkh T., Lee K., Kim Y.J. (2012). Investigations of transported and local emissions on particle compositions in Korea. Aerosol Air Qual. Res..

[10] Park S., Jung S., Gong B., Cho S., Lee S. (2013). Characteristics of PM_2.5_ haze episodes revealed by highly time-resolved measurements at an air pollution monitoring supersite in Korea. Aerosol Air Qual. Res..

[11] Dons E., Panis L.I., van Poppel M., Wets G. (2012). Personal exposure to black carbon in transport microenvironments. Atmos. Environ..

[12] Hagemann R., Corsmeier U., Kottmeier C., Rayk R., Wieser A., Vogel B. (2014). Spatial variability of particle number concentrations and NO_x_ in the Karlsruhe (Germany) area obtained with the mobile laboratory ‘AERO-TRAM’. Atmos. Environ..

[13] Krecl P., Johansson C., Strom J., Lovenheim B., Gallet J.C. (2014). A feasibility study of mapping light-absorbing carbon using a taxi fleet as a mobile platform. Tellus B.

[14] Pattinson W., Longley I., Kingham S. (2014). Using mobile monitoring to visualize diurnal variations of traffic pollutants across two near-highway neighbourhoods. Atmos. Environ..

[15] Zhu Y., Hinds W.C., Kim S., Sioutas C. (2002). Concentration and size distribution of ultrafine particles near a major highway. J. Air Waste Manag. Assoc..

[16] Franklin J.E., Drummond J.R., Griffin D., Pierce J.R., Waugh D.L., Palmer P.I., Parrington M.P., Lee J.D., Lewis A.C., Rickard A.R. (2014). A case study of aerosol depletion in a biomass burning plume over Eastern Canada during the 2011 BORTAS field experiment. Atmos. Chem. Phys..

[17] KORUS-AQ, Data Archive, an International Cooperative Air Quality Field Study in Korea. https://www-air.larc.nasa.gov/missions/korus-aq/.

[18] Wu Y., Xia X. (2016). Effect of ambient humidity on the light absorption amplification of black carbon in Beijing during January 2013. Atmos. Environ..

[19] Yun D., Kim M., Lee J., Kim B., Lee D., Lee S., Yu S., Kim S. (2015). Correction factors for outdoor concentrations of PM_2.5_ measured with portable real-time monitors compared with gravimetric methods: Results from South Korea. J. Environ. Sci. Int..

[20] Kim S., Wipfli H., Navas-Acien A., Dominici F., Avila-Tang E., Onicescu G., Breysse P., Samet J.M. (2009). Determinants of hair nicotine concentrations in nonsmoking women and children: A multicountry study of secondhand smoke exposure in homes. Cancer Epidemiol. Biomark. Prev..

[21] Targino A.C., Gibson M.D., Krecl P., Rodrigues M.V., Dos Santos M.M., de Paula Corrêa M. (2016). Hotspots of black carbon and PM_2.5_ in an urban area and relationships to traffic characteristics. Environ. Pollut..

[22] Bilkis A.B., Anwar H., Nurun N., Andreas M., Philip K.H. (2012). Organic and Black Carbon in PM_2.5_ at an Urban Site at Dhaka, Bangladesh. Aerosol Air Qual. Res..

[23] Ahlm L., Liu S., Day D.A., Russell L.M., Weber R., Gentner D.R., Goldstein A.H., Keutsch F.N., VandenBoer T.C., Markovic M.Z. (2012). Formation and growth of ultrafine particles from secondary sources in Bakersfield, California. J. Geophys. Res..

[24] Vilcassim M.J.R., Thurston G.D., Peltier R.E., Gordon T. (2014). Black Carbon and Particulate Matter (PM_2.5_) Concentrations in New York City’s Subway Stations. Environ. Sci. Technol..

[25] Park S., Lee K. (2015). Characterization and sources of black carbon in PM_2.5_ at a site close to a roadway in Gwangju, Korea, during winter. Environ. Sci. Process. Impacts.

[26] Mar T.F., Norris G.A., Koenig J.Q., Larson T.V. (2000). Associations between air pollution and mortality in Phoenix, 1995–1997. Environ. Health Perspect..

[27] Ostro B., Feng W.Y., Broadwin R., Green S., Lipsett M. (2007). The effects of components of fine particulate air pollution on mortality in California: Results from CALFINE. Environ. Health Perspect..

[28] Klemm R.J., Lipfert F.W., Wyzga R.E., Gust C. (2004). Daily mortality and air pollution in Atlanta: Two years of data from ARIES. Inhal. Toxicol..

[29] Cakmak S., Dales R.E., Vida C.B. (2009). Components of particulate air pollution and mortality in Chile. Int. J. Occup. Environ. Health.

